# Parliament and Publications

**Published:** 1911-05-27

**Authors:** 


### Parliament and Publications.

### MEDICAL INSPECTION OF SCHOOL CHILDREN.

Replying to a question, Mr. Runciman said that medical officers for the inspection of children in public elementary schools are paid by the local education authorities who appoint them. There is no uniform rate of payment, and he was unable to say whether the Local Government Board in Ireland was making arrangements for putting the order for the inspection of school children in force.

### NAVAL MEDICAL SERVICE.

Lord Charles Beresford asked the First Lord of the Admiralty whether there was a deficiency of sixty-six surgeons on the establishment for those officers in the Navy list; whether the number of ships in commission would probably be increased, and what steps the Admiralty intended to take to fill these vacancies? Mr. McKenna replied that the number of surgeons was at present fifty-four below the authorised establishment. It was hoped that the recommendations of the committee on the naval medical service, when carried into effect, would enable the deficiency to be made good.

### LUNACY LEGISLATION.

The Lord Chancellor has introduced in the House of Lords a Bill to amalgamate the lunacy departments and to transfer the power of making Vesting Orders from the Judge in Lunacy to the High Court. It also proposes to add two more medical men to the Commissioners. The Commissioners in Lunacy, whose numbers remain the same as they were in 1845, though the number of lunatics under their care has increased from 25,000 to 120,000, have for several years urgently represented that their numbers are insufficient. The Royal Commission, which reported in 1908, went very fully into the subject, and made an emphatic recommendation that two additional medical commissioners should be appointed at once. The scheme provides that the unpaid commissioners may include women.

